# Isoliensinine exerts antitumor effects in lung adenocarcinoma by inhibiting APEX1-driven ROS production

**DOI:** 10.3389/fphar.2025.1555802

**Published:** 2025-05-27

**Authors:** Cheng Chen, Chenying Shu, Dan Shen, Zhaowei Yan, Zeyi Liu, Jian-An Huang

**Affiliations:** ^1^ Department of Pulmonary and Critical Care Medicine, the First Affiliated Hospital of Soochow University, Suzhou, China; ^2^ Institute of Respiratory Diseases, Soochow University, Suzhou, China; ^3^ Suzhou Key Laboratory for Respiratory Diseases, Suzhou, China; ^4^ Department of Anesthesiology, Zhejiang Cancer Hospital, Hangzhou, Zhejiang, China; ^5^ Department of Pharmacy, The First Affiliated Hospital of Soochow University, Suzhou, China

**Keywords:** Isoliensinine, lung adenocarcinoma, APEX1, reactive oxygen species, natural compound

## Abstract

**Introduction:**

Lung cancer is considered to be the world’s leading malignancy in morbidity and mortality, and despite great efforts to treat it no effective treatment has yet been found. Isoliensinine is a natural small-molecule drug with potent anti-tumor activity in several cancer cell lines. Here, we have shown that Isoliensinine exhibits anti-tumor activity against lung adenocarcinoma (LUAD) both *in vitro* and *in vivo*.

**Methods:**

The biological functions of Isoliensinine in LUAD cells were investigated using CCK8 assay, colony formation, transwell assays and flow cytometry assays. DARTS assay was used to validate Isoliensinine targets screened by site prediction and molecular docking. The *in vivo* anti-tumor efficacy of Isoliensinine was analyzed in the xenograft tumor model.

**Results:**

The IC_50_ of Isoliensinine were 6.98 μM, 17.24 μM and 16.00 μM in H1299, A549, H1650 cell lines while 28.65 μM in BEAS-2B cells. Isoliensinine inhibits the proliferation, migration, invasion of LUAD cells and arrests the cell cycle and promotes LUAD cells apoptosis *in vitro*. Isoliensinine attenuates tumor growth in a murine xenograft mode. Mechanistically, Isoliensinine interacted directly with APEX1, inhibited APEX1 protein levels, and promoted ROS generation. Knockdown of APEX1 reverses the effect of Isoliensinine on LUAD cells.

**Discussion:**

Isoliensinine exerts antitumor effects through inhibition of APEX1 driven ROS production, therefore, Isoliensinine may represent a new drug candidate to be used to treat LUAD.

## 1 Introduction

Lung cancer is the world’s leading malignancy in morbidity and mortality (Thandra et al., 2021). Lung adenocarcinoma (LUAD) accounts for about 45% of the newly diagnosed cases (Sung et al., 2021). The molecular pathogenesis of lung cancer is quite complex and heterogeneous, and can be caused by a variety of genetic factors and epigenetic changes (e.g., amplifications, point mutations, deletions, insertions and translocations) (Dakal et al., 2024; Smolarz et al., 2025). Lung cancer is an insidious disease, and many patients are already in mid- or late-stage disease when they are found, missing the best opportunity for surgery. For patients with advanced NSCLC, commonly used treatments include chemotherapy, radiotherapy (Zhu et al., 2022), targeted therapy (Guo H. et al., 2022) and immunotherapy (Desai and Peters, 2023). Notwithstanding the plethora of options available, the prognosis for lung cancer remains poor, with a low 5-year survival rate. Consequently, there is an ongoing need to research new drugs to treat lung cancer. For thousands of years, natural compounds have always been one of the main sources of new drugs, which are a class of highly promising drugs with wide sources and relatively low toxic side effects (Katz and Baltz, 2016).

Isoliensinine is a natural compound with the chemical formula C_37_H_42_N_2_O_6_ and molecular weight: 610.7392. Isoliensinine can be extracted from all parts of the lotus root (Zhao et al., 2014). Regarding its anti-tumor mechanism, Law et al. found that Isoliensinine induced various types of cellular autophagy, including PC-3, MCF-7, A549, H1299, Hep3B, and LO2 cells, and he also found that Isoliensinine induced autophagy through the activation of the AMPK/TSC2/mTOR signaling pathway, which induced apoptosis, in cervical cancer (Law et al., 2014). Zhang et al. found that in triple-negative breast cancer, Isoliensinine induced apoptosis through the p38 MAPK/JNK pathway (Zhang et al., 2015). Shu et al. found that Isoliensinine triggered apoptosis in HCC cells by inducing p65 dephosphorylation and inhibiting NF-κB (Shu et al., 2016). Li et al. found that Isoliensinine induced cell cycle arrest and apoptosis in cervical cancer cells through inhibition of the AKT/GSK3α pathway (Li et al., 2022). Xu et al. reported that Isoliensinine induced UBQLN1-mediated stabilisation of PGC1α overcomes hypoxia-induced drug resistance in hepatocellular carcinoma cells (Xu et al., 2025). Wu et al. found that Isoliensinine can affect mitochondrial autophagy as a therapeutic strategy for renal cell carcinoma (Wu et al., 2025). Prasath found that Isoliensinine combined with platinum in the treatment of colorectal cancer enhanced ROS-mediated endogenous apoptosis by activating the MAPK/PI3K/AKT pathway (Manogaran et al., 2019), and he also found that cisplatin resistance was reversed in colorectal cancer stem cells (Manogaran et al., 2022). Hu et al. reported that Isoliensinine can inhibit the proliferation and migration of gastric cancer cells by targeting TGFBR1 and regulating the TGF-β-smad signalling pathway (Hu et al., 2024).

Although Isoliensinine has been reported to have antitumor activity in a wide variety of tumors, whether to treat lung adenocarcinoma has not yet been reported. Therefore, the present study aimed to explore the antitumor effects and possible mechanisms of Isoliensinine in LUAD cells.

## 2 Materials and methods

### 2.1 Materials

BEAS-2B, H1299, A549, and H1650 cells were purchased from the Cell Bank of the Chinese Academy of Sciences (Shanghai, China). Cells were cultured using RPMI 1640 medium (Gibco, Carlsbad, CA, United States) containing 10% fetal bovine serum at 37°C and 5% CO_2_ atmosphere. Isoliensinine was supplied by Shanghai Yuanye Technology Co., Ltd. with a purity >95%.

### 2.2 Cell counting kit-8(CCK-8) assays

100 μL of cell suspension containing 3,000 cells was inoculated into 96-well plates and incubated for 24–48 h until the cells reached 40%–50% of the plate bottom. The original medium was removed and a fresh medium containing Isoliensinine (1–50 μmol) was added and incubated for 48 h 10 μL CCK-8/well (Sigma-Aldrich, St. Louis, MO, United States) was added to incubate the cells for 2–3 h. The absorbance of the orange formazan was then read and quantified.

### 2.3 Colony formation

After treatment with Isoliensinine for 48 h, 3,000 cells were counted and seeded in 60 mm dishes containing complete medium and cultured until lesion formation, then washed with PBS, fixed with methanol, and stained with crystal violet (Beyotime, Shanghai, China).

### 2.4 Migration and invasion transwell assay

In the Transwell assay, tumor cells were pre-treated with Isoliensinine for 48 h. Matrigel matrix (BD Science, Sparks, MD, United States) was applied to the upper chamber for invasive assays. The cells (2 × 10^4^ cells for migration assay and 3 × 10^4^ cells for invasion assay) were seeded in the upper chamber of Transwell inserts containing 1% FBS medium. Added 800 ul complete medium to the lower chamber. After incubation, tumor cells that had migrated to the lower layer were fixed with methanol and stained with crystal violet (Beyotime, Shanghai, China). Finally, it was observed and photographed using a light microscope.

### 2.5 Cell cycle and apoptosis analysis

Cell cycle and apoptosis analysis kit purchased from Beyotime Biotechnology (Shanghai, China). For cell cycle assays, cells were incubated in 70% ethanol at 4°C overnight and then stained with a mixture of PI and RNaseA for 30 min at 37°C in the dark. For the apoptosis assay, cells were stained with PI and Annexin V/FITC. FACSCalibur (Beckman Coulter, Brea, CA, United States) was used to detect stained cells.

### 2.6 Target prediction

The targets of Isoliensinine were predicted using the SuperPred database. The database is available at https://prediction.charite.de/index.php.

### 2.7 Molecular docking

Molecular docking of predicted targets with Isoliensinine was conducted using AutoDock-vina (version 1.5.6) software. Low binding affinity values indicate a stronger and more stable binding between the receptor and ligand.

### 2.8 DARTS assay

Cell lysates were prepared with lysis buffer (0.4% Tritonx-100, 400 mM NaCl, 20% glycerol, 100 mM Tris-HCL (pH = 7.5)). Cell lysates were mixed with DMSO and different concentrations of Isoliensinine overnight at 4°C. Protease was added to hydrolyze the proteins for 10 min and reaction was terminated by adding 5x SDS-PAGE loading buffer followed by Western blotting.

### 2.9 Western blot analysis

The antibodies used in the analysis were anti-APEX1 (Proteintech Group, Inc., China), anti-N-cadherin, anti-E-cadherin (BD Biosciences, Sparks, MD, United States), anti-cyclin D1 (Abcam, London, United Kingdom), anti-tubulin, anti-Lamin-B1 (Proteintech Group, Inc., China), anti-PARP, anti-β-actin, anti-mouse and anti-rabbit secondary antibodies (Cell Signaling Technology, Danvers, MA, United States).

### 2.10 Cellular fractionation

The nuclear protein extraction kit was purchased from TransGen Biotech (DE201-01, TransGen Biotech). Cells were lysed using a Cytoplasmic ProteinExtract Buffer, and nuclear proteins were extracted using Nuclear Extract Buffer.

### 2.11 Immunofluorescence

After treatment with Isoliensinine for 48 h, the cells were cultured on coverslips. Cell fixation was performed with 4% formaldehyde for 20 min and permeabilization with 0.5% Triton X-100 PBS solution for 20 min at 20°C. After blocking with 5% BSA for 1 h, the cells were incubated with anti-APEX1 overnight at 4°C and appropriate Cy3-labelled secondary antibody for 1 h in the dark. Nuclei were stained with DAPI for 5 min and captured by confocal microscope.

### 2.12 Patient samples

The experiments were approved by the Ethics Committee of the First Affiliated Hospital of Soochow University (Application approval number: 2020–375). All experiments were performed in accordance with relevant guidelines and regulations. We confirm that informed consent was obtained from all subjects and/or their legal guardian(s).

With the approval of the Ethics Committee of the First Affiliated Hospital of Soochow University, a total of 20 paired fresh LUAD and paracancerous tissues were collected from 2022 to 2023 to make pathological cut edges. Patients did not receive chemotherapy or radiotherapy.

### 2.13 Immunohistochemical (IHC) analysis

Sections were incubated with anti-APEX1 (1:100 dilution; Proteintech Group, Inc., China) overnight at 4°C and secondary antibodies for 1 h. The reactions were developed with DAB (BD Bioscience, San Jose, CA, United States) and counterstained with hematoxylin.

### 2.14 Reactive oxygen species (ROS) assay

ROS was detected ROS assay kit (Beyotime Biotechnology, China). After treatment with Isoliensinine for 48 h, cells were incubated with DCFH-DA at 37°C for 30 min and analyzed by flow cytometry.

### 2.15 Transfection with shRNA

The viral transfection solution used to interfere with APEX1 was provided by Shanghai Genechem Co., Ltd. Target short hairpin RNA (shRNA): 5′-CAG​AGA​AAT​CTG​CAT​TCT​ATT-3′. Cells were seeded into 6-well plates, Add the mixed viral transfection solution and incubated at 37°C in a 5% CO_2_ incubator. 20 h later, replace to normal medium. Use puromycin screening for 2 weeks to purify the transfected successful cell lines.

### 2.16 *In vivo* tumor xenograft animal model

The animal experiment was approved by the Ethics Committee of First Affiliated Hospital of Soochow University. BALB/c nude mice (Female, 5 weeks old) were obtained from the Experimental Animal Center of Soochow University. The flanks were inoculated subcutaneously with a total of 2.5 × 10^6^ A549 cells. Twelve mice were randomly divided into two groups: a castor oil control group and an Isoliensinine group (20 mg/kg). When the tumor weight reached nearly 100 mm^3^, the mice were given an intraperitoneal injection of castor oil or Isoliensinine every 2 days for a total of 7 times. Tumor growth was evaluated by volume (V = L (tumor length) × W^2^ (tumor width)/2). When the largest tumor volume reached 1000 mm^3^, all mice euthanized by cervical dislocation. Tumors were removed and weighed. Half of the tumors were pathologically sectioned for immunohistochemistry, and the other half of the tumors were subjected to protein extraction by Western blot analysis.

The animal study protocol was approved by the Ethics Committee of the First Affiliated Hospital of Soochow University. All experiments were performed in accordance with relevant guidelines and regulations. The study was reported in accordance with ARRIVE guidelines (https://arriveguidelines.org). Mice were euthanized via cervical dislocation.

### 2.17 Statistical analysis

All experiments were repeated three times. All results are presented as the means ± SD (standard deviation). Statistical comparisons were determined with the Student’s test, and *p*-value was regarded as significant. All statistical analyses were performed with GraphPad Prism 8.0 (GraphPad, San Diego, CA) and SPSS 25.0 software (SPSS, Chicago, IL).

## 3 Results

### 3.1 Isoliensinine inhibits the proliferation, migration, and invasion of LUAD cells *in vitro*

**FIGURE 1:**
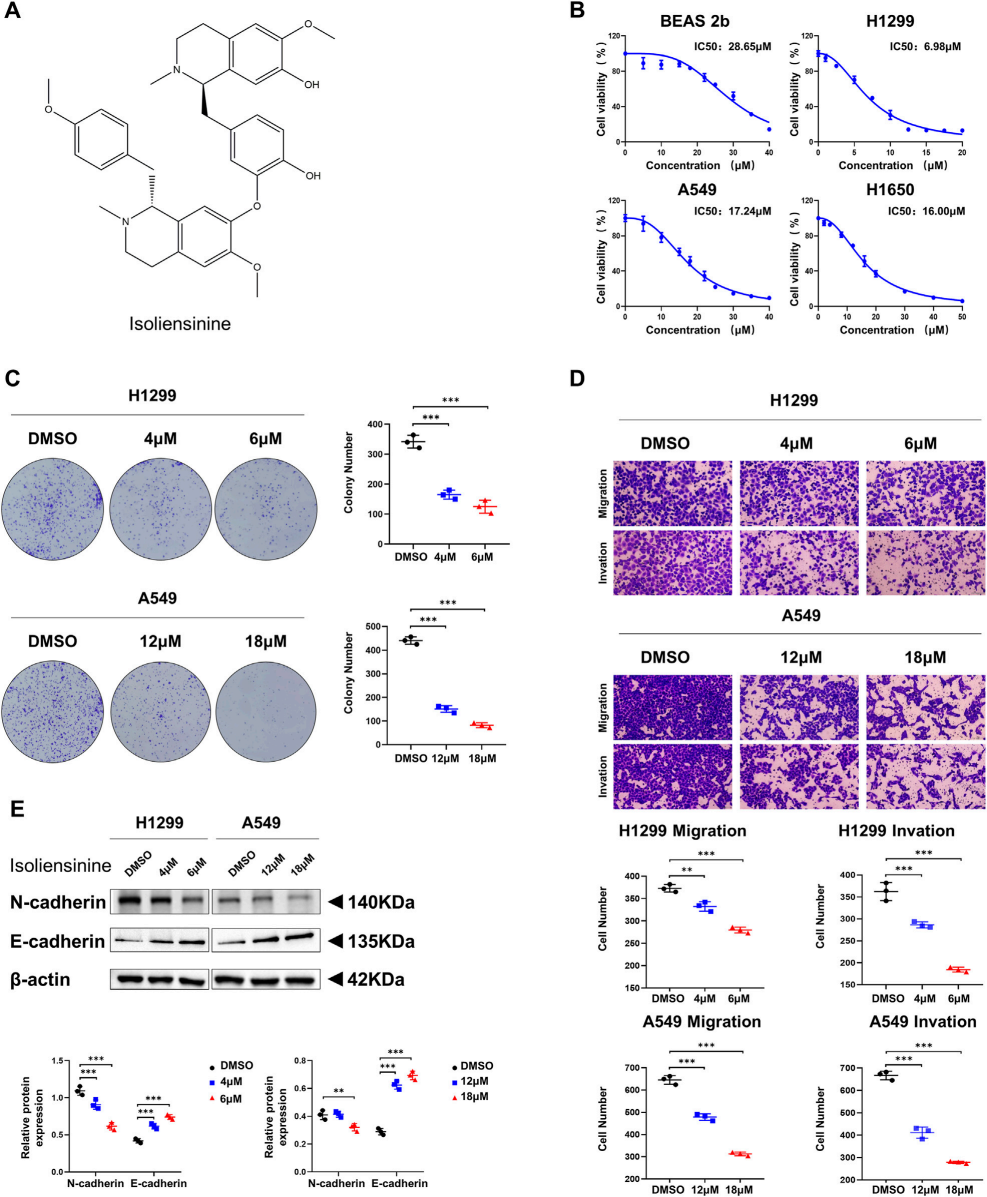
Isoliensinine inhibits the proliferation, migration and invasion of LUAD cells *in vitro*. **(A)** Chemical structure of Isoliensinine. **(B)** BEAS 2b, H1299, A549 and H1650 cells were treated with the indicated concentrations of Isoliensinine for 48 h. Cell viability was determined by cck-8 assay. The IC_50_ of Isoliensinine for each cell line was calculated according to a cell viability value. **(C)** Representative images of the results of the clonogenic analysis of H1299 and A549 cell proliferation after treatment with or without Isoliensinine. **(D)** Representative images showing the results of Transwell cell migration and invasion assays of H1299 and A549 cells treated with or without Isoliensinine. **(E)** The protein expression of N-cadherin and E-cadherin in H1299 and A549 cells treated with or without Isoliensinine as determined by Western blot analysis. Student’s t-test was used for statistical analysis and data were presented as the mean ± SD. * *p*-value <0.05; ** *p*-value <0.01; *** *p*-value <0.001.

The Chemical structure of Isoliensinine was shown in Figure 1A. CCK-8 assay showed substantial declines in cell viability after treatment with Isoliensinine for 48 h. In LUAD cells, the half-maximal inhibitory concentration (IC_50_) of Isoliensinine on cell viability was lower than bronchial epithelial BEAS-2B cell. The IC_50_ of Isoliensinine were 6.98 μM, 17.24 μM and 16.00 μM in H1299, A549, H1650 cell lines while 28.65 μM in BEAS-2B cells (Figure 1B). Clone formation experiments showed that the proliferative capacity of Isoliensinine-treated tumor cells was lower than that of untreated cells (Figure 1C). Transwell assay showed a dose-dependent inhibition of migration and invasion of H1299 and A549 cells after treatment with Isoliensinine (Figure 1D). We investigated the potential mechanism of Isoliensinine to inhibit metastasis and proliferation by Western blotting, and found that after cells were treated with Isoliensinine, the expression of N-cadherin, an EMT-related marker, was decreased, while E-cadherin was increased (Figure 1E).

### 3.2 Isoliensinine arrests the cell cycle and promotes LUAD cells apoptosis

**FIGURE 2:**
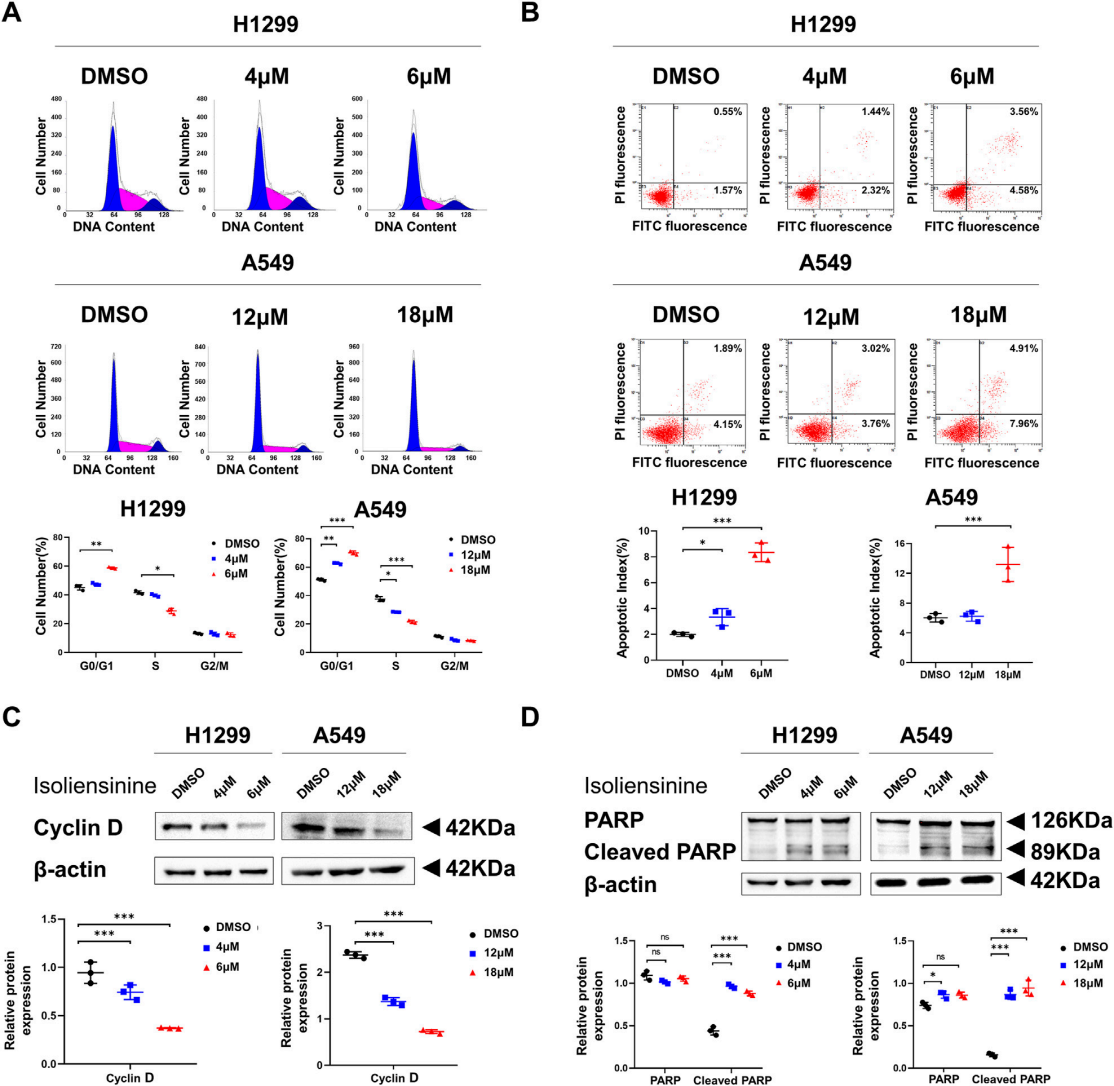
Isoliensinine arrests the cell cycle and promotes the apoptosis. **(A)** H1299 and A549 were harvested at 48 h after treatment with or without Isoliensinine and stained with PI. The percentage of cells in each cell cycle phase is shown in the inset of each panel. **(B)** H1299 and A549 harvested at 48 h after transfection and stained with Annexin V-FITC and PI. The right histogram panel shows the statistics of the number of apoptotic cells in each group. **(C,D)** The protein expression of cyclin D, PARP and cleaved PARP in H1299 and A549 cells treated with or without Isoliensinine as determined by Western blot analysis. Student’s t-test was used for statistical analysis and data were presented as the mean ± SD. * *p*-value <0.05; *** *p*-value <0.001 vs. the control.

The proportion of cells treated with Isoliensinine in the G0/G1 phase increased, while the S phase decreased compared to cells not treated with Isoliensinine (Figure 2A). The results of flow cytometry also indicated an increase in apoptosis among the cells treated with Isoliensinine. (Figure 2B). The cycle-associated protein Cyclin D and the apoptosis-associated protein cleaved PARP were also changed (Figures 2C,D). The effect of Isoliensinine treatment on cell cycle and apoptosis was concentration-dependent.

Using RNA-Seq to detect gene transcriptional profiles in H1299 cells after the addition of Isoliensinine and functional enrichment analyses, it was found that the biological functions of Isoliensinine can affect cell proliferation, migration, cycling, and apoptosis (Supplementary Figure S1).

### 3.3 Isoliensinine can interact with apurinic/apyrimidinic endodeoxyribonuclease 1 (APEX1, APE1, or REF1) directly

**FIGURE 3:**
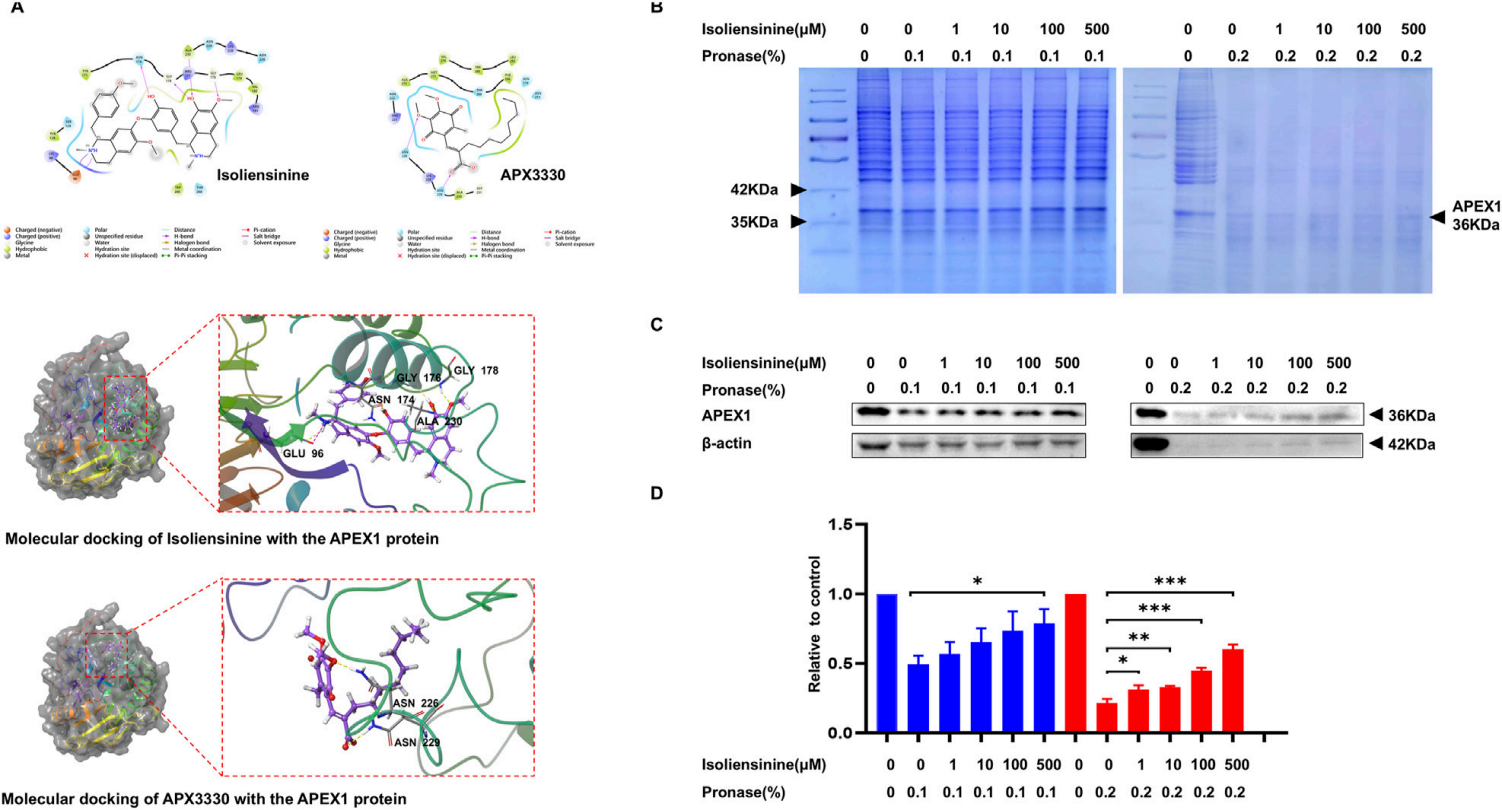
Isoliensinine interacts with APEX1 directly. **(A)** Molecular docking conformation of Isoliensinine with the APEX1 protein, the MM-GBSA results was −39.27 kcal/mol. Molecular docking conformation of APX3330 with the APEX1 protein, the MM-GBSA results was −27.27 kcal/mol. **(B–D)** Cell lysates were mixed with DMSO and different concentrations of Isoliensinine overnight at 4°C. Protease was added to hydrolyse the proteins for 10 min and the reaction was terminated by the addition of 5x SDS-PAGE loading buffer. **(B)** The SDS-PAGE gels were stained by coomassie blue. **(C–D)** The content of APEX1 was analyzed by APEX1 antibody. * *p*-value <0.05, ** *p*-value <0.01, *** *p*-value <0.001 versus control.

We used the SuperPred database to predict the target of Isoliensinine. Autodock vina 1.5.6 was used for molecular docking of Isoliensinine and target proteins (Supplementary Table S1). The high affinity between Isoliensinine and APEX1 (PDB ID: 6BOW). Isoliensinine binds to the surface of the active pocket of APEX1 protein, and APEX1 protein residues TRP280, VAL180, and LEU179 form a hydrophobic force on Isoliensinine. The ligand forms a hydrogen bond with each of residues GLY178, ALA230, GLY176, and ASN174, and a hydrogen bond and a salt bridge with residue GLU96. The classical APEX1 inhibitor APX3330 binds to the surface of the active pocket of the APEX1 protein, and APEX1 protein residues PHE266, LEU282, and TRP280 form a hydrophobic force on APX3330. The ligand forms a hydrogen bond with each of residues ASN226 and ASN229. There are some differences in the regions where Isoliensinine and APX3330 bind to the APEX1 protein, with Isoliensinine covering a large range of the protein activity pocket and APX3330 only binding to the upper range of the protein activity pocket. Molecular docking conformation of Isoliensinine with the APEX1 protein, the MM-GBSA results was −39.27 kcal/mol. Molecular docking conformation of APX3330 with the APEX1 protein, the MM-GBSA results was −27.27 kcal/mol. In comparison, Isoliensinine binds more stably to APEX1 (Figure 3A). DARTS assay has been widely used for the identification of drug targets (Ren et al., 2021). Isoliensinine-treated lanes had higher band intensities between 35 and 42 kD than Isoliensinine-untreated lanes (Figure 3B). Western blot later confirmed that the band was APEX1 (Figures 3C,D) suggesting that Isoliensinine binds to APEX1.

### 3.4 APEX1 expression is increased in tumor tissues

**FIGURE 4:**
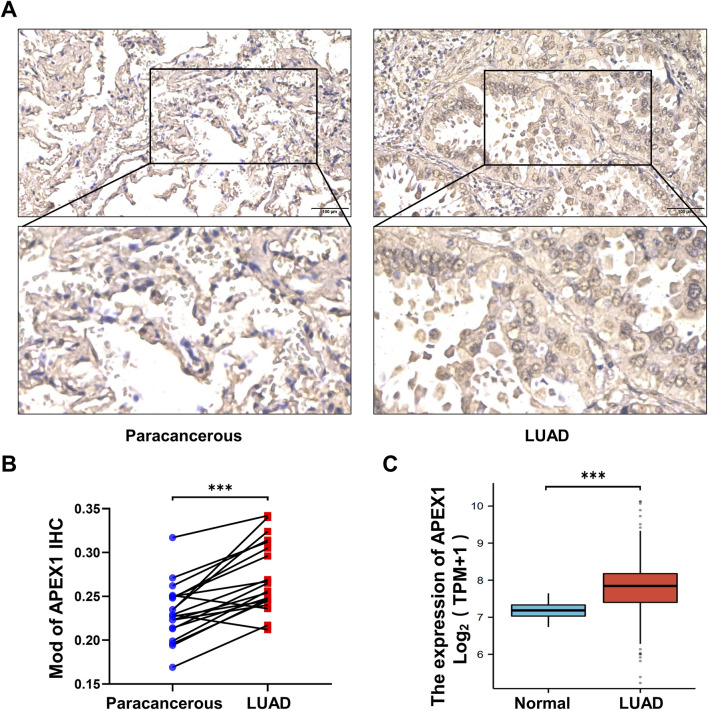
APEX1 expression is increased in LUAD. **(A,B)** Immunohistochemical detection of APEX1 expression in LUAD tumor tissues and paracancerous tissues. **(C)** APEX1 expression in normal and LUAD patients in the TCGA database. *** *p*-value <0.001.

We collected tumor tissues and paracancerous tissues from 20 patients with pathologically confirmed LUAD and detected APEX1 expression by immunohistochemistry (Supplementary Table S2; Supplementary Figure S2). The results confirmed that APEX1 expression was higher in tumor tissues than in paracancerous tissues (Figures 4A,B). In addition, the expression of APEX1 in LUAD patients included in the TCGA database was also higher than that in normal patients (Figure 4C). These results demonstrate that APEX1 can be a target for the treatment of LUAD.

### 3.5 Isoliensinine reduces APEX1 protein and promotes reactive oxygen species (ROS) production

**FIGURE 5:**
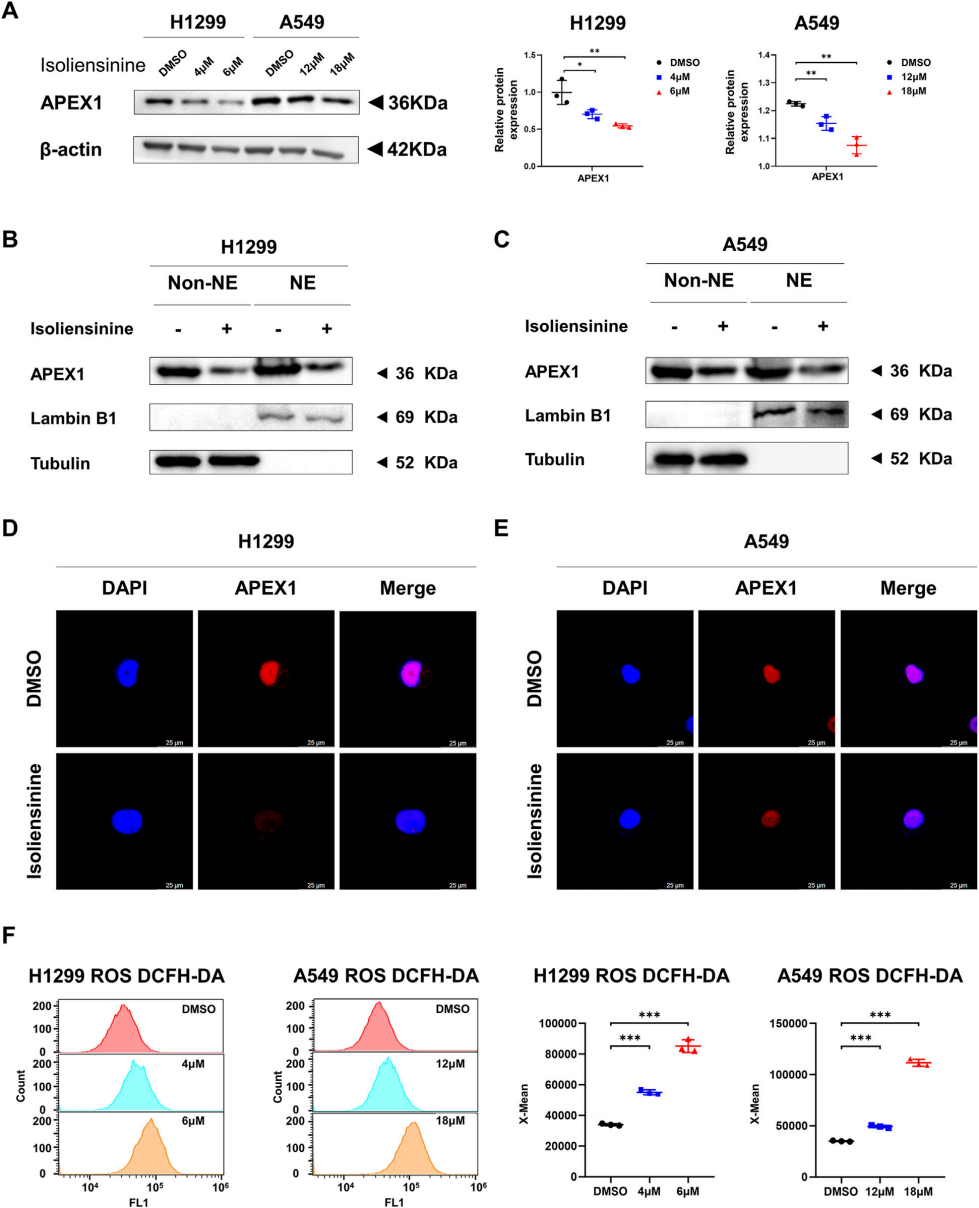
Isoliensinine suppresses APEX1 protein expression and promotes ROS generation. **(A)** The protein expression of APEX1 in H1299 and A549 cells treated with or without Isoliensinine as determined by Western blot analysis. **(B,C)** H1299 and A549 cells treated with or without Isoliensinine subject to cellular fractionation followed by Western blotting for detecting APEX1. Tubulin and Lamin B1 are endogenous markers of cytosolic and nuclear proteins (NE: Nuclear, Non-NE: Cytoplasmic). **(D–E)** Immunofluorescence staining of APEX1 after A549 and H1299 cells were treated with or without Isoliensinine. **(F)** Determination of ROS by flow cytometry assay in H1299 and A549 cells treated with or without Isoliensinine. * *p*-value <0.05, ** *p*-value <0.01, *** *p*-value <0.001.

Western blot was used to examine the effect of Isoliensinine on APEX1 protein, and it was found that Isoliensinine reduced the protein level of APEX1 in a dose-dependent manner (Figure 5A). As APEX1 is translocated to the nucleus and serves as a redox signaling hub for the regulation of transcription factors, we verified the effects of Isoliensinine on APEX1 expression in the cytoplasm (non-NE) and nucleus (NE) by Cellular fractionation assay and Immunofluorescence assay. The results showed that the content of APEX1 in both cytoplasm and nucleus was decreased in the tumor cells treated with Isoliensinine (Figures 5B-E). As APEX1 is an important reduction factor, we further examined ROS content. It was found that ROS levels increased with the use of Isoliensinine (Figure 5F).

### 3.6 The effects of Isoliensinine on LUAD cells could be reversed by knockdown of APEX1

**FIGURE 6:**
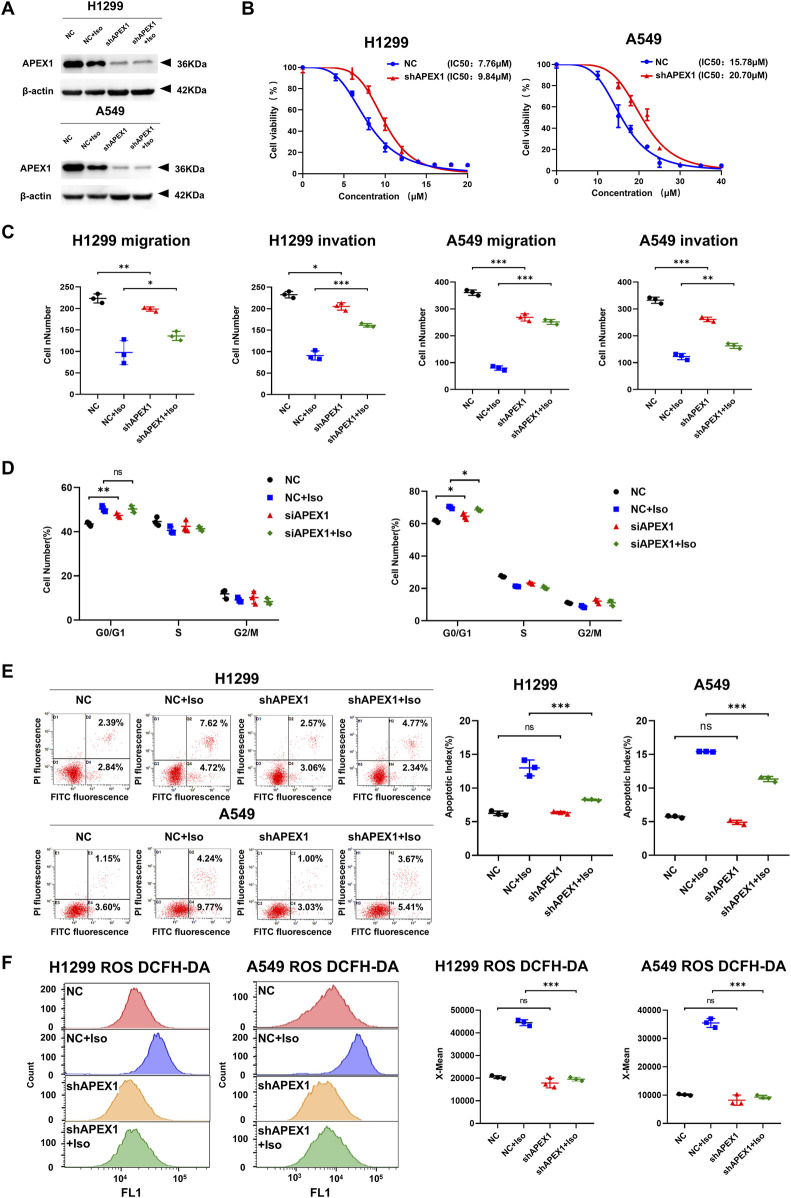
The effects of Isoliensinine on LUAD cells could be reversed by knockdown of APEX1. **(A)** The protein expression of shAPEX1. **(B)** The viability of shAPEX1 H1299 and A549 cells after treated with Isoliensinine was determined by cck-8 assay. **(C)** Transwell assay to detect the migration and invasion ability of cells and shAPEX1 cells treated with or without Isoliensinine. **(D–F)** Flow cytometry assay to detect the cell cycle, apoptosis and ROS of cells and shAPEX1 cells treated with or without Isoliensinine. Ns no significance, * *p*-value <0.05, ** *p*-value <0.01, *** *p*-value <0.001.

As Isoliensinine can interact directly with APEX1, we knocked down APEX1 (Figure 6A) and again performed the CCK8 assay, Transwell assays, and flow cytometry assays. It was found that the effects of Isoliensinine on H1299 and A549 cells were reversed after the knockdown of APEX1 (Figures 6B–E; (Supplementary Figure S4). The effect of Isoliensinine on ROS was also reversed (Figure 6F; Supplementary Figure S3).

### 3.7 Isoliensinine attenuates tumor growth in a murine xenograft mode

**FIGURE 7:**
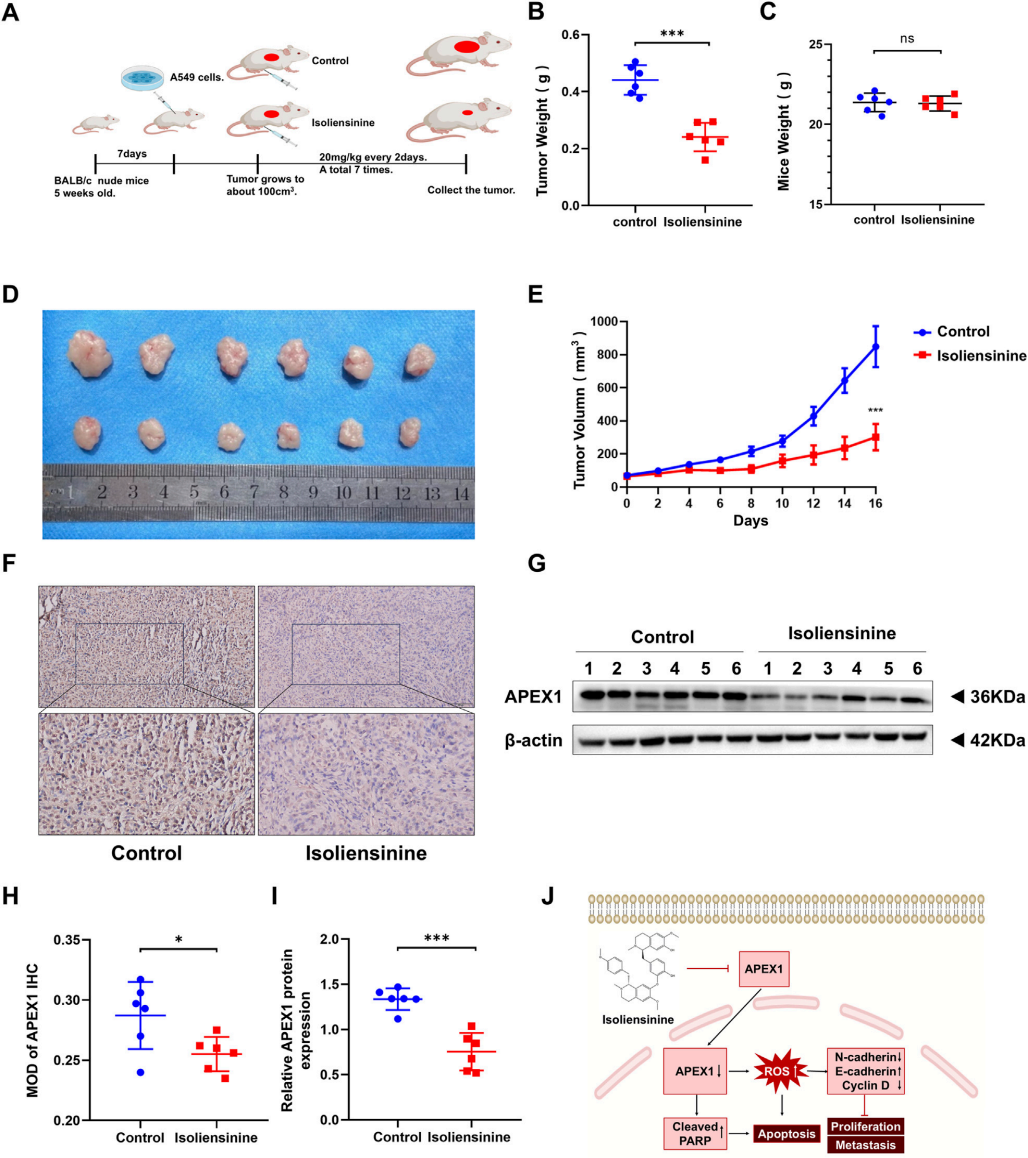
Isoliensinine attenuates tumor growth in a murine xenograft model. **(A)** A549 cell xenografts in nude mice (n = 6 in each group). Isoliensinine at a concentration of 20 mg/kg was injected intraperitoneally every 2 days, and tumors were harvested. **(B)** Tumor weight. **(C)** Mice weight. **(D)** Morphology of tumor. **(E)** Tumor volume. **(F,H)** Immunohistochemical detection of APEX1 expression. **(G,I)** Western blotting analysis of APEX1 protein expression in tumor tissues. **(J)** Hypothetical model showing how Isoliensinine acts in LUAD cells. * *p*-value <0.05, ** *p*-value <0.01, *** *p*-value <0.001.

We further assessed the role of Isoliensinine *in vivo*. A549 cells were injected into BALB/c mice. Isoliensinine (20 mg/kg) was injected intraperitoneally every 2 days (Figure 7A). It was found that the tumor volume and weight were lower in the Isoliensinine-treated group than in the control group (Figures 7B–D). After 7 sessions, we executed the mice and removed the xenograft tumors. We detected APEX1 expression in the tumors by immunohistochemistry and Western blotting. The results all confirmed that the expression of APEX1 in the Isoliensinine treatment group was lower than that in the control group (Figures 7E–H). These results confirmed that Isoliensinine can inhibit tumor growth by suppressing the expression of APEX1 *in vivo*.

Taken together, these data indicate that Isoliensinine inhibits LUAD cell proliferation, migration, and invasion arrests the cell cycle, and promotes LUAD cells apoptosis mainly by reducing the APEX1 target protein.

## 4 Discussion

The antitumor effects of Isoliensinine have now been reported in a variety of tumor therapies (Li et al., 2022; Manogaran et al., 2022; Manogaran et al., 2023; Liu et al., 2021). There are no studies on the role of Isoliensinine in LUAD. Our study was the first investigation of the antitumor role of Isoliensinine in LUAD cells. We demonstrated that Isoliensinine inhibited the proliferation, migration, and invasive ability of LUAD cells. Isoliensinine also induced LUAD cell cycle arrest and apoptosis. We used the superpred database to predict the target of Isoliensinine. Autodock vina 1.5.6 was used for molecular docking of Isoliensinine and target proteins so that target proteins with better affinity for Isoliensinine could be selected and validated with DARTS experiments. We finally confirmed that APEX1 is the target protein of Isoliensinine.

APEX1 is an endothelial deoxyribonuclease required for the base excision repair (BER) pathway that maintains genome stability (Allinson et al., 2004) and also serves as a redox signaling hub for the regulation of transcription factors (Antoniali et al., 2017; Malfatti et al., 2020; Malfatti et al., 2021). APEX1 regulates signal transduction and activates many transcription factors such as activator protein 1 (AP-1), nuclear factor (NF)κB, transcription 3 (STAT3), p53, and hypoxia-inducible factor (HIF)-1α (Logsdon et al., 2016; Li et al., 2019; Siqueira et al., 2024; Fishel et al., 2011). Thus, APEX1 has emerged as an attractive target in tumors.

Numerous studies have reported overexpression of APEX1 in non-small cell lung cancer (NSCLC) (Gu et al., 2013; Wei et al., 2016). High APEX1 expression is associated with poor prognosis, aggressiveness, resistance to radiotherapy, resistance to targeted therapy, and efficacy of immunological drugs. Increased APEX1 expression causes NF-κB activation and contributes to poor prognosis in NSCLC (Wu et al., 2010; Wu et al., 2013). APEX1 is elevated after treatment with platinum-based drugs, and elevated levels correlate with poor OS (Zhang et al., 2016). APEX1 targets the STING pathway, protecting LUAD cells from radiation damage and inducing radiation resistance (Zhou et al., 2023). Mutations in key epidermal growth factor receptor (EGFR) driver genes can affect downstream molecular networks and pathways, thereby influencing therapeutic response in NSCLC, and IPA causal network analysis revealed that the causal network of APEX1 was highly activated under the Ex19del mutation (Nishimura et al., 2020). NSCLC patients with EGFR mutations with low APEX1 expression achieved longer PFS and OS time after treatment with EGFR-TKI, and APEX1 protein levels were significantly elevated in EGFR-TKI-resistant cell lines, APEX1 activates Akt signaling in lung adenocarcinoma through a redox-dependent mechanism, which stimulates epidermal growth factor receptor-TKI resistance, a specific APEX1 inhibitor (APX3330) can make cells more sensitive to EGFR-TKI (Lu et al., 2018; Yang et al., 2018). Pre-treatment APEX1 levels correlate with the clinical outcomes of patients with advanced NSCLC treated with immune checkpoint inhibitors (ICIs) monotherapy and combination therapy, and patients with higher pre-treatment APEX1 levels had shorter PFS times, both with ICIs monotherapy and combination therapy (Hu et al., 2023).

We investigated the immunohistochemical levels of APEX1 in cancer and paracancerous tissues of 20 patients with pathologically confirmed LUAD and confirmed that the APEX1 level in tumor tissue was higher than that in paracancerous tissue, which is in line with other studies reported (Gu et al., 2013; Wei et al., 2016). We found that Isoliensinine could target and downregulate the protein level of APEX1 both *in vivo* and *in vitro*. APEX1 mediated hypoxia-induced reactive oxygen species (ROS) generation. Elevated ROS exacerbates oxidative damage to all important macromolecules (e.g., proteins, fats, carbohydrates, etc.), leading to irreversible cellular damage and cell death (Jelic et al., 2021; Kuo et al., 2022). There have been many studies demonstrating that tumor cell death can be promoted by increasing ROS affecting physiological processes in tumor cells (Wang et al., 2021). Indeed many commonly used chemotherapeutic agents such as cisplatin and 5-fluorouracil also kill tumor cells by directly or indirectly promoting the accumulation of ROS (Zaidieh et al., 2019; Rawat et al., 2020). APEX1 acts as a redox signaling hub that can regulate ROS (Liu et al., 2020), and drugs that inhibit APEX1 have been shown to promote ROS generation to exert anticancer effects (Ullah et al., 2021). Elevated ROS were observed in cells treated with Isoliensinine. The effect of Isoliensinine on ROS decreased after APEX1 was knocked out, demonstrating that the effect of allantoin on ROS is mediated through APEX1.

Ferroptosis is a recently discovered type of programmed cell death. HIF-1α, Nrf2, ROS and p53 pathway have been demonstrated to be involved in ferroptosis. APEX1 is closely related to these pathways. This makes it possible to modulate ferroptosis by APEX1 inducers or inhibitors as a treatment for various human diseases (Guo N. et al., 2022). APEX1 can treat cancer by affecting ferroptosis has been reported in a variety of tumors (e.g., hepatocellular carcinoma (Du et al., 2024; Diao et al., 2024), gastric cancer (Zhao et al., 2023), osteosarcoma (Xiao et al., 2024), etc.). In this study, we confirmed that Isoliensinine directly inhibits APEX1. Therefore, Isoliensinine could also affect ferroptosis. In recent years, Isoliensinine has been reported to ferroptosis death in other diseases (Long et al., 2024; Song et al., 2025). Therefore, in future study, we will further explore whether Isoliensinine can affect ferroptosis in LUAD. In addition, since ferroptosis is involved in the resistance to targeted drugs in lung cancer, we will further explore whether Isoliensinine can reverse the resistance to targeted drugs.

## 5 Conclusion

This study identified the antitumor functions of Isoliensinine in LUAD cells. In addition, we demonstrated that Isoliensinine regulates the cell cycle and apoptosis by inhibiting APEX1, thereby promoting the generation of ROS to exert anti-tumor effects. These features suggest that Isoliensinine has an anticancer effect on LUAD. Isoliensinine may represent a new drug candidate to be used to treat LUAD.

## Data Availability

The original contributions presented in the study are publicly available. This data can be found here: [https://db.cngb.org/ [accession number CNP0007390].

## Supplementary material

SUPPLEMENTARY FIGURE S1 Pathways enriched for gene expression changes occurring in the Isoliensinine treatment group compared to the control group. **(A)** Top20 of Go Enrichment. **(B)** Pathway analysis.

SUPPLEMENTARY FIGURE S2 Immunohistochemical of APEX1 in tumor and paracancerous tissues of LUAD patients.

SUPPLEMENTARY FIGURE S3 Isoliensinine promotes ROS generation. **(A)** ROS in H1299 and A549 cells treated with or without Isoliensinine. **(B)** ROS of cells and shAPEX1 cells treated with or without Isoliensinine.

SUPPLEMENTARY FIGURE S4 The effects of Isoliensinine on LUAD cells could be reversed by knockdown of APEX1. **(A)** Representative images showing the results of Transwell cell migration and invasion assays of cells and shAPEX1 cells treated with or without Isoliensinine. **(B)** Flow cytometry assay to detect the cell cycle of cells and shAPEX1 cells treated with or without Isoliensinine.

SUPPLEMENTARY FIGURE S5 The IC_50_ of Isoliensinine for each cell line in different time point.
