# Supplementary material for: Isoliensinine exerts antitumor effects in lung adenocarcinoma by inhibiting APEX1-driven ROS production
Source: Front Pharmacol. 2025 May 27;16:1555802. doi: 10.3389/fphar.2025.1555802 (PMC12149194; doi:10.3389/fphar.2025.1555802)
Supplement: Supplementary file 1 [file Table1.docx]

| **Table1： SUPERPred predicted targets for Isoliensinine (Top 10)** | | | | |
| --- | --- | --- | --- | --- |
| **Target Name** | **UniProt ID** | **PDB Visualization** | **Probability** | **Model accuracy** |
| Nuclear factor NF-kappa-B p105 subunit（NF-kappa-B p105） | P19838 | 1SVC | 99% | 96% |
| Hypoxia-inducible factor 1 alpha（HIF 1α） | Q16665 | 4H6J | 97% | 85% |
| Proteasome component C5 | P20618 | 6KWY | 94% | 90% |
| DNA-(apurinic or apyrimidinic site) lyase（APEX1） | P27695 | 6BOW | 94% | 91% |
| Transcription intermediary factor 1-alpha（TIF 1α） | O15164 | 4YBM | 94% | 96% |
| NT-3 growth factor receptor （NGFR） | Q16288 | 6KZD | 94% | 96% |
| Cathepsin D | P07339 | 4OD9 | 93% | 99% |
| Glutamate NMDA receptor; GRIN1/GRIN2B | Q05586 | 5EWM | 92% | 96% |
| Protein tyrosine kinase 2 beta | Q14289 | 4EKU | 92% | 91% |
| Histone deacetylase 8 | Q9BY41 | 5VI6 | 91% | 94% |
