# Supplementary figures and images for: Isoliensinine exerts antitumor effects in lung adenocarcinoma by inhibiting APEX1-driven ROS production
Source: Front Pharmacol. 2025 May 27;16:1555802. doi: 10.3389/fphar.2025.1555802 (PMC12149194; doi:10.3389/fphar.2025.1555802)

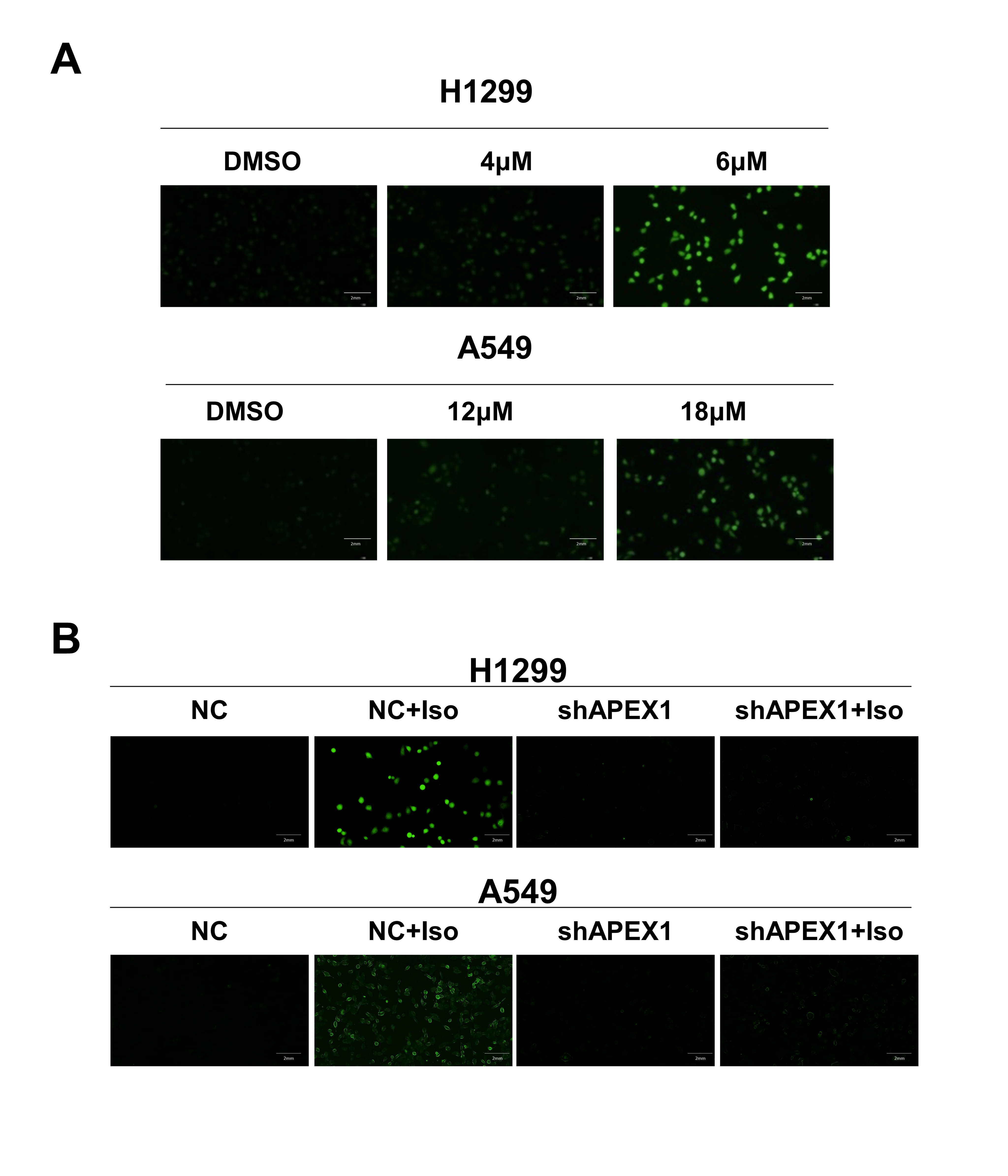

Supplement: Supplementary file 2 [file Image3.tif]

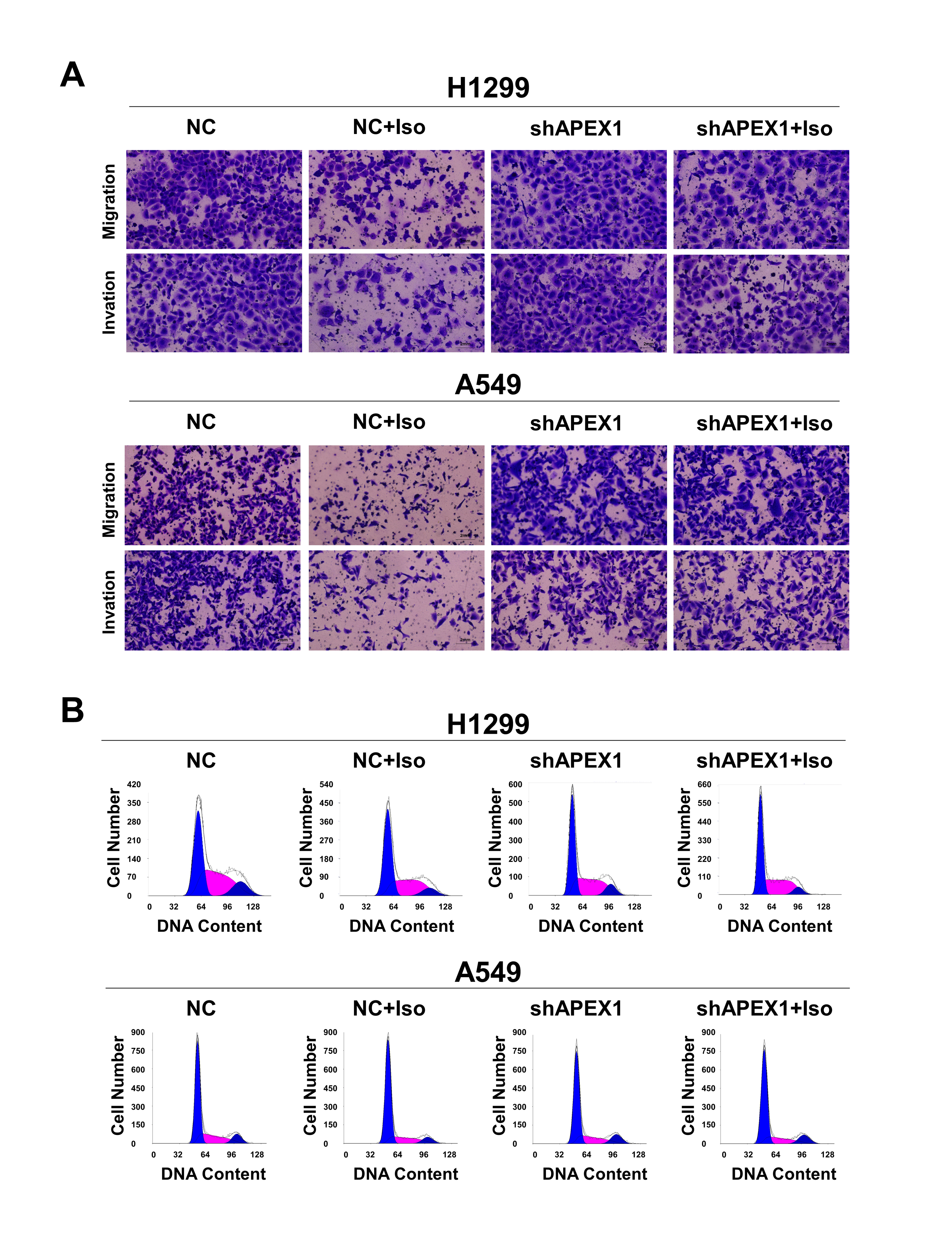

Supplement: Supplementary file 3 [file Image4.tif]

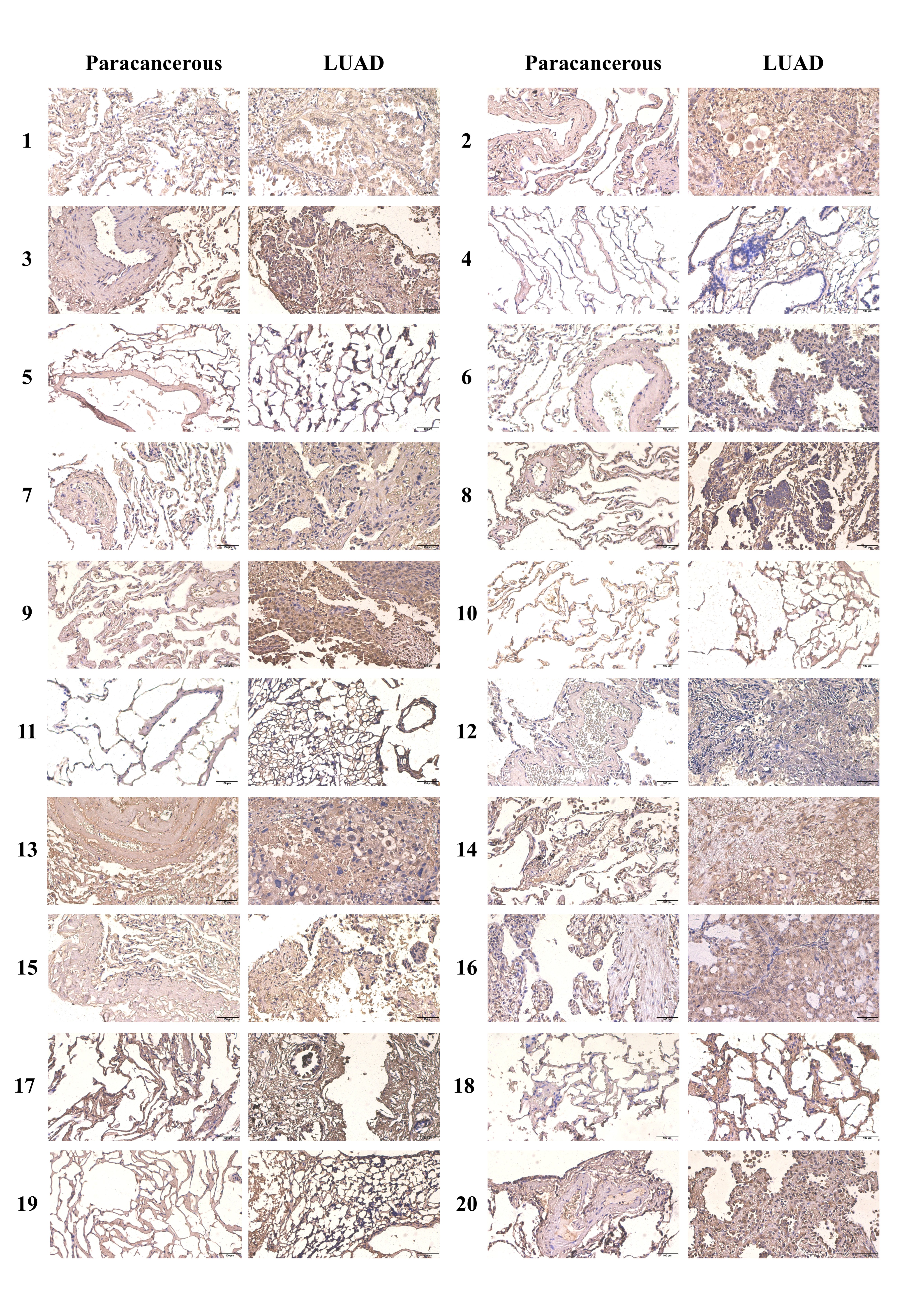

Supplement: Supplementary file 4 [file Image2.jpeg]

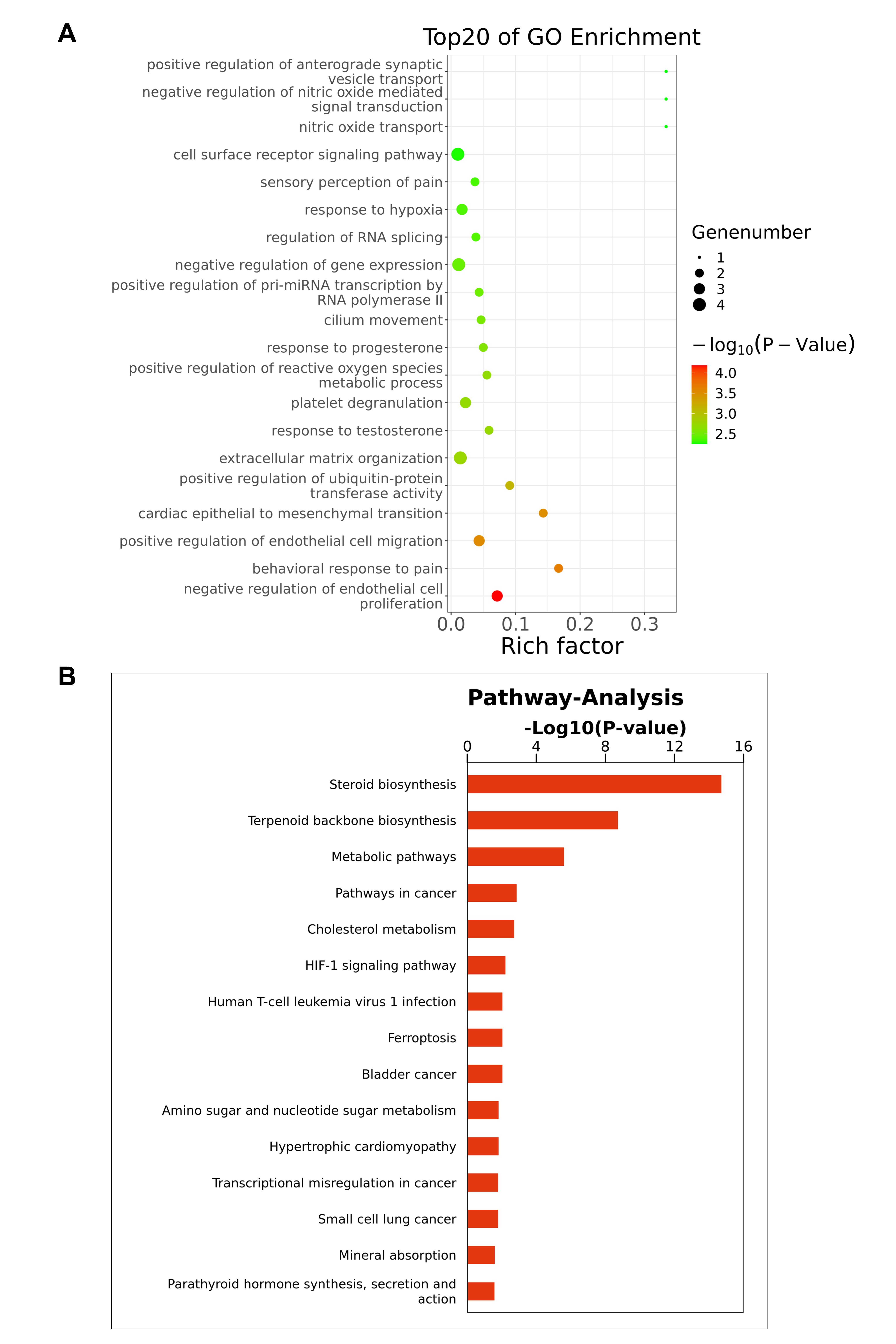

Supplement: Supplementary file 5 [file Image1.tif]

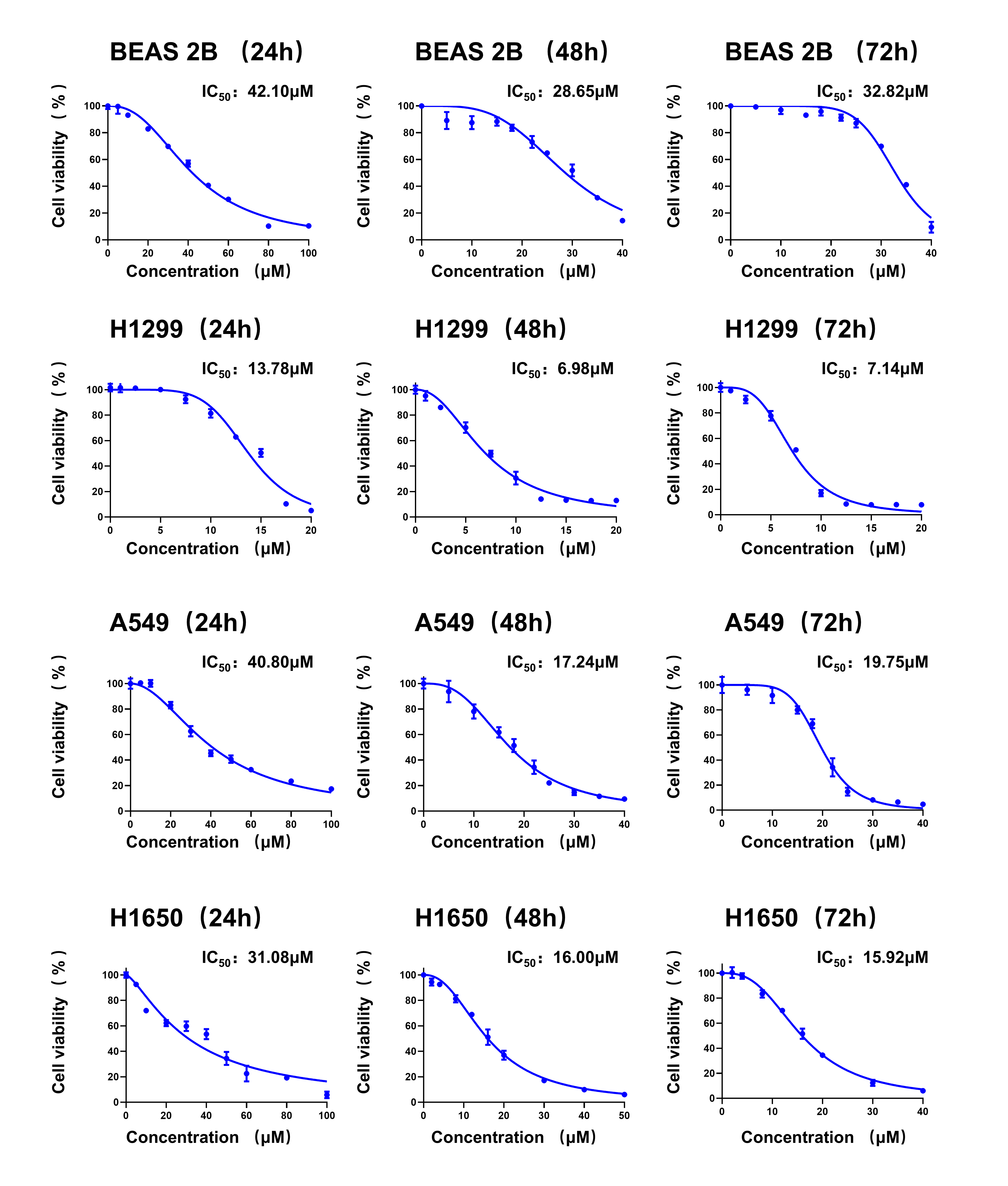

Supplement: Supplementary file 7 [file Image5.tif]
