# Supplementary material for: Isoliensinine exerts antitumor effects in lung adenocarcinoma by inhibiting APEX1-driven ROS production
Source: Front Pharmacol. 2025 May 27;16:1555802. doi: 10.3389/fphar.2025.1555802 (PMC12149194; doi:10.3389/fphar.2025.1555802)
Supplement: Supplementary file 6 [file Table2.docx]

| **Basic patient information** | | | |
| --- | --- | --- | --- |
| **Patient** | **Gender** | **Age** | **Tumour TNM staging** |
| 1 | Male | 54 | IB (T2aN0M0) |
| 2 | Female | 83 | IIA (T1N1M0) |
| 3 | Female | 61 | IIB (T3N0M0) |
| 4 | Female | 57 | IA (T1bN0M0) |
| 5 | Female | 63 | IIA (T1bN1M0) |
| 6 | Male | 69 | IB (T2aN0M0) |
| 7 | Male | 76 | IA (T1bN0M0) |
| 8 | Female | 58 | IA (T1bN0M0) |
| 9 | Female | 61 | IA (T1bN0M0) |
| 10 | Male | 65 | IA (T1bN0M0) |
| 11 | Male | 48 | IIB (T3N0M0) |
| 12 | Female | 41 | IA (T1aN0M0) |
| 13 | Male | 71 | IIIA (T4N0M0) |
| 14 | Male | 70 | IIIA (T1cN2bM0) |
| 15 | Male | 74 | IIB (T2bN1M0) |
| 16 | Male | 69 | IA (T1bN0M0) |
| 17 | Male | 59 | IA (T1bN0M0) |
| 18 | Female | 62 | IA (T1cN0M0) |
| 19 | Male | 63 | IB (T2aN0M0) |
| 20 | Female | 56 | IA (T1bN0M0) |
